# Sex-Based Differences in Long-Term Outcomes Following Percutaneous Coronary Intervention for Chronic Total Occlusions

**DOI:** 10.3390/jcm15041449

**Published:** 2026-02-12

**Authors:** Ignacio Gallo, Rafael Gonzalez-Manzanares, Luis Carlos Maestre-Luque, Francisco Hidalgo, Guillermo Soriano, Cristina Urbano, Javier Suárez de Lezo, José Segura-Aumente, Gloria Heredia, Diana Ladera, Miguel Romero, Manuel Pan, Soledad Ojeda

**Affiliations:** 1 Department of Cardiology, Reina Sofia University Hospital, 14002 Córdoba, Spain; z22gafei@uco.es (I.G.); h42malul@uco.es (L.C.M.-L.); francisco.hidalgo.sspa@juntadeandalucia.es (F.H.); cristina.urbano.sspa@juntadeandalucia.es (C.U.); javier.suarez.sspa@juntadeandalucia.es (J.S.d.L.); jmaria.segura.sppa@juntadeandalucia.es (J.S.-A.); gloriam.heredia.sspa@juntadeandalucia.es (G.H.); maria.diana.ladera.sspa@juntadeandalucia.es (D.L.); miguel.romero.moreno.sspa@juntadeandalucia.es (M.R.); manuel.pan.sspa@juntadeandalucia.es (M.P.); sopineda@uco.es (S.O.); 2 Maimonides Biomedical Research Institute of Cordoba (IMIBIC), 14002 Córdoba, Spain; guillermo.soriano@imibic.org; 3 Department of Medical and Surgical Sciences, University of Cordoba, 14002 Córdoba, Spain; 4 Centro de Investigación Biomédica en Red Enfermedades Cardiovasculares (CIBERCV), 28029 Madrid, Spain

**Keywords:** chronic total occlusion, sex differences, percutaneous coronary intervention

## Abstract

**Background/Objectives**: Sex-based differences in clinical profiles and outcomes following percutaneous coronary intervention (PCI) for chronic total occlusions (CTO) remain poorly understood. We sought to examine the association between sex and long-term clinical outcomes following CTO-PCI in a contemporary real-world cohort. **Methods**: We conducted a retrospective study of 928 consecutive patients (788 men, 140 women) undergoing CTO-PCI at a high-volume centre between 2011 and 2024. The primary endpoint was a composite of major adverse cardiac events (MACE: all-cause death, myocardial infarction [MI], or stroke) at a 6-year follow-up. To account for baseline differences, an Inverse Probability of Treatment Weighting (IPTW)-adjusted Cox regression analysis was performed. **Results**: Women were significantly older (69.7 ± 10 vs. 64.1 ± 10 years; *p* < 0.001) and had a higher prevalence of diabetes and hypertension. However, women exhibited lower angiographic complexity, with lower J-CTO scores (2 [1–2] vs. 2 [1–3]; *p* < 0.001) and less frequent severe calcification or tortuosity. Technical and procedural success rates were comparable between sexes (85.4% vs. 86.7%; *p* = 0.695). Unadjusted MACE rates were higher in women (29.3% vs. 22.1%; hazard ratio (HR) 1.51, 95% CI: 1.08–2.13; *p* = 0.017). After adjustment, the female sex was no longer associated with the primary endpoint (aHR 1.15, 95% CI: 0.76–1.74; *p* = 0.517), but the risk of MI remained significantly higher in this group (aHR 2.85, 95% CI: 1.23–6.63; *p* = 0.015). **Conclusions**: CTO-PCI appeared to be equally safe and effective in women and men. Over long-term follow-up, although the overall adjusted MACE risk was similar between sexes, the female sex was associated with a higher risk for MI.

## 1. Introduction

Cardiovascular disease is the leading cause of mortality in women, who exhibit distinct clinical characteristics, risk profiles, and prognoses compared with men [1]. In the setting of coronary artery disease (CAD), women generally present with less extensive atherosclerosis and a lower burden of diffuse and complex subclinical disease compared with men [2]. Despite these differences, women remain underrepresented in most clinical trials and registries and are often undertreated in routine practice [3,4,5]. When women undergo CTO revascularization, prior studies suggest worse procedural outcomes, including higher complication rates, adverse in-hospital events, and mortality during follow-up [6,7,8]. Indeed, the female sex has been identified as an independent predictor of procedural complications in validated CTO risk scores, such as the PROGRESS-CTO complication score, reflecting the combined influence of anatomical, physiological, and comorbidity-related factors.

Given the reported sex-related disparities in periprocedural outcomes and the limited evidence regarding long-term clinical outcomes following CTO-PCI, a detailed characterization of sex-specific outcomes is warranted [9]. Our study aims to address this gap by analysing sex-based differences in long-term outcomes after CTO-PCI in a real-world setting, providing insights that may inform and optimize clinical decision-making in the management of women with CTO.

## 2. Materials and Methods

### 2.1. Study Design and Setting

We conducted an observational, longitudinal, retrospective study of all consecutive patients who underwent percutaneous coronary intervention (PCI) for chronic total occlusions (CTO) at a high-volume tertiary centre (Reina Sofia University Hospital, Córdoba, Spain) between January 2011 and 2024. The study protocol was approved by the Clinical Research Ethics Committee of Córdoba (Spain) (identification number SICEIA-2025-003513) and was conducted in accordance with the Declaration of Helsinki and Good Clinical Practice guidelines. Due to the retrospective nature of the study and the use of de-identified data, the requirement for written informed consent was waived by the institutional review board.

### 2.2. Population

The study population included all patients ≥ 18 years of age with a diagnosis of at least one CTO who underwent a PCI attempt during the study period. Indications for CTO-PCI were established by a multidisciplinary Heart Team. Patients were stratified according to sex to evaluate potential disparities in baseline clinical characteristics, angiographic complexity, procedural strategies, and long-term outcomes between women and men. Clinical data, angiographic parameters, and procedural details were gathered from a prospectively maintained institutional database. Follow-up outcomes were reviewed from electronic medical records. Follow-up duration was truncated at 6 years or at the time of death, whichever occurred first.

### 2.3. Definitions and Endpoints

A CTO was defined as the presence of Thrombolysis In Myocardial Infarction (TIMI) flow grade 0 within the occluded segment for an estimated duration of ≥3 months [10]. Angiographic complexity was assessed using the SYNTAX score and the J-CTO (Multicentre CTO Registry in Japan) score [11,12].

The primary endpoint was the incidence of major adverse cardiovascular events (MACE) at long-term follow-up, defined as a composite of all-cause death, myocardial infarction (MI), or stroke. Secondary endpoints included the individual components of the primary endpoint and clinically driven revascularization.

Procedural outcomes were defined according to the CTO Academic Research Consortium (CTO-ARC) consensus [10]. Technical success was defined as the achievement of TIMI flow grade 3 with <30% residual stenosis in the target lesion. Procedural success was defined as technical success in the absence of in-hospital major adverse cardiovascular events.

MI was defined according to the Fourth Universal Definition of MI and CTO-ARC criteria. Periprocedural MI (Type 4a) required a cTn elevation >5 times the 99th percentile of the upper reference limit, along with evidence of new ischemia. Long-term MI events during the study period were collected from electronic health records and independently reviewed by two board-certified cardiologists. Acute kidney injury (AKI) and major bleeding were classified according to the CTO-ARC definition and BARC type 3a or higher, respectively [10,13].

### 2.4. Statistical Analysis

Categorical data were presented as counts (percentages) and continuous data as mean ± standard deviation or median (interquartile range). Between-group comparisons were made using the chi-square test or the Fisher exact test for categorical variables and the Student *t*-test or the Mann–Whitney U-test for continuous variables as appropriate. For the clinical endpoints analysis, unadjusted and Inverse Probability of Treatment Weighting (IPTW)-adjusted Cox proportional hazards regression models were used to calculate hazard ratios (HRs) with 95% confidence intervals (CIs). For non-fatal endpoints, estimates corresponded to cause-specific HRs, where patients who died without experiencing the event of interest were censored at the time of death. To account for the competing risk of death, sensitivity analyses were performed using Fine-Gray subdistribution hazard models. The proportional hazards assumption was verified using Schoenfeld residuals. For the IPTW-adjusted analyses, propensity scores were estimated using a logistic regression model with female sex as the dependent variable and the following covariates: age, diabetes mellitus, hypertension, atrial fibrillation, chronic obstructive pulmonary disease (COPD), prior CAD, left ventricular ejection fraction (LVEF), estimated glomerular filtration rate (eGFR), acute coronary syndrome (ACS), left main disease, left anterior descending (LAD) artery involvement, J-CTO score, and multivessel disease. Weights were stabilized to reduce the influence of extreme values. Covariate balance before and after weighting was assessed using Standardized Mean Differences (SMDs), with an SMD < 15% being considered indicative of adequate balance. As the percentage of missingness was <5% for baseline data, missing values were handled using a single imputation for IPTW analysis. To ensure the robustness of our results, a sensitivity analysis was performed using multiple imputation of covariates across 20 datasets followed by a ‘within-imputation’ approach using the MatchThem package (version 1.2.1) [14]. All statistical analyses were performed using R software (version 4.4.2; R Foundation for Statistical Computing, Vienna, Austria), and a 2-sided *p*-value < 0.05 was considered statistically significant.

## 3. Results

### 3.1. Baseline Clinical Characteristics

A total of 928 patients were included, consisting of 788 (85%) men and 140 (15%) women. Female patients were significantly older than male patients (69.7 ± 10.0 vs. 64.1 ± 10 years; *p* < 0.001). Regarding the cardiovascular risk profile, women had a higher prevalence of hypertension (74.3% vs. 60.0%; *p* = 0.002) and diabetes mellitus (59.0% vs. 43.7%; *p* < 0.001) but were less frequently active smokers (20.3% vs. 33.8%; *p* = 0.002) and had a lower prevalence of COPD (4.3% vs. 9.9%; *p* = 0.048). Women were less likely to have prior coronary artery disease (29.0% vs. 40.4%; *p* = 0.017) and prior PCI (30.4% vs. 41.2%; *p* = 0.024). Baseline LVEF was higher in female patients compared to male patients (55.9 ± 11.1% vs. 52.0 ± 12.3%; *p* = 0.001) (Table 1).

### 3.2. Angiographic and Procedural Characteristics

Data from 1003 CTO lesions were analysed (Table 2). Women presented with significantly lower anatomical complexity: shorter lesion lengths (25 [18–38] mm vs. 35 [21–50] mm; *p* < 0.001), shorter CTO segments (18 [13–25] mm vs. 22 [16–34] mm vs; *p* < 0.001), and lower incidences of severe calcification (47.2% vs. 58.1%; *p* = 0.019), severe tortuosity (8.7% vs. 15.8%; *p* = 0.042), and CTO bifurcations (23.3% vs. 33.6%; *p* = 0.018). Accordingly, the median J-CTO score was lower in female patients (2 [1–2] vs. 2 [1–3]; *p* < 0.001).

Procedural strategies also differed by sex (Table 2). Femoral artery access was less frequently utilized as the primary approach in women compared to men (49.3% vs. 72.6%; *p* < 0.001). Anterograde wire escalation was utilized more frequently in women (82.5% vs. 67.4%; *p* < 0.001), whereas retrograde wire escalation was less common (5.0% vs. 11.2%; *p* = 0.027). Intravascular ultrasound (IVUS) was less frequently employed in women (25.4% vs. 16.4%; *p* = 0.025). Procedures in women were associated with shorter procedural times (101.8 ± 48.3 vs. 134.7 ± 79.6 min; *p* < 0.001), contrast volumes (234.7 ± 154.4 vs. 252.7 ± 111.1 mL; *p* = 0.014), and radiation exposure levels (Air Kerma: 1592 (1049–2810) vs. 2297 (1395–3454) mGy; *p* = 0.001). No significant sex-based differences were observed in technical success (86.7% vs. 85.4%; *p* = 0.695) or procedural success (86.5% vs. 85.4%; *p* = 0.794) groups.

### 3.3. In-Hospital Complications

The incidence of in-hospital adverse events was low and comparable between sexes (Table 3). There were no significant differences in rates of in-hospital death (0.7% vs. 0.1%; *p* = 0.270), coronary perforation (1.4% vs. 1.0%; *p* = 0.667), or cardiac tamponade (1.4% vs. 0.5%; *p* = 0.213).

### 3.4. Long-Term Clinical Outcomes

**Figure 1:**
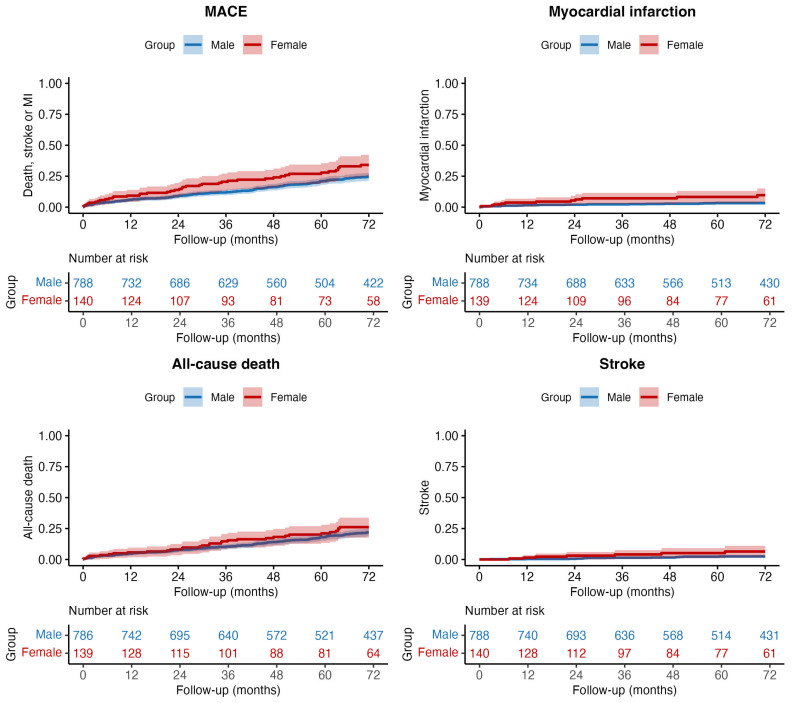
6 years follow up Kaplan–Meier curves of the outcomes. MACE: Major adverse cardiovascular events.

After a median follow-up 72 (IQR 43–72) months, the primary endpoint of MACE occurred in 215 (23.2%) patients: 41 (29.3%) women and 174 (22.1%) men (unadjusted HR 1.51, 95% CI: 1.08–2.13; *p* = 0.017) (Figure 1). The association between the female sex and an increased risk of MACE was driven by the occurrence of higher rates of MI and stroke in women compared to men (Table 4), but there were no differences in all-cause mortality.

**Figure 2:**
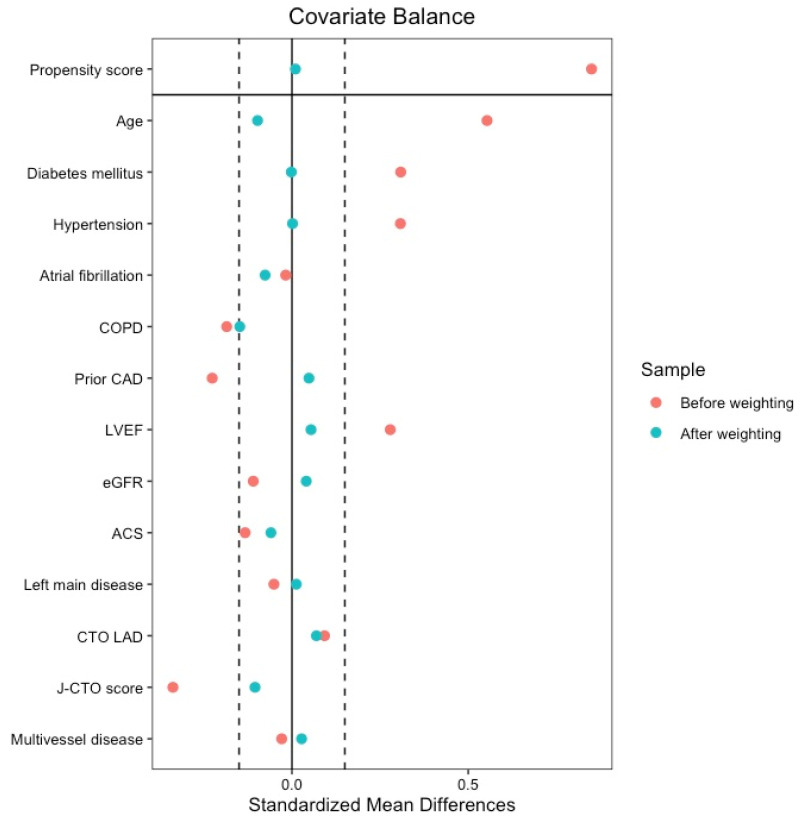
Balance of the covariates before and after weighting. ACS: acute coronary syndrome; CAD: coronary artery disease; COPD: chronic obstructive pulmonary disease; CTO: chronic total occlusion; eGFR: estimated glomerular filtration rate; LAD: left anterior descending artery; and LVEF: left ventricular ejection fraction.

To account for baseline differences, Cox models were adjusted using IPTW. In the weighted sample, sex groups showed a good balance for all the included covariates, with all SMDs being below 0.15 (Figure 2). The performance of the propensity model was assessed through the c-statistic, which was 0.735 (95% CI, 0.690–0.779), indicating an appropriate discriminatory capacity. Furthermore, the distribution of propensity scores showed substantial overlap between groups after weighting, as displayed in Supplementary Figure S1. In the IPTW-adjusted MACE Cox model, the association between sex and the primary endpoint was no longer significant (aHR 1.15, 95% CI: 0.76–1.74; *p* = 0.517). However, the female sex remained independently associated with a significantly higher risk of MI (aHR 2.85, 95% CI: 1.23–6.63; *p* = 0.015). Fine-Gray models for non-fatal events showed consistent findings, including a higher risk of MI in women even after accounting for the competing risk of death (sHR 2.88, 95% CI: 1.24–6.68; *p* = 0.014 (Supplementary Table S1). No significant differences were found in the adjusted rates of all-cause death (aHR 0.90; *p* = 0.646) or stroke (aHR 1.95; *p* = 0.193). A summary of clinical endpoints rates and measures of associations is presented in Table 4. The estimates were consistent in a sensitivity analysis handling missing covariate data using multiple imputations (Supplementary Table S2 and Figure S2).

## 4. Discussion

The main findings of our study were as follows: (1) women presented with a distinct clinical and angiographic phenotype characterized by a higher burden of baseline comorbidities despite significantly lower lesion complexity compared to men; (2) technical and procedural success rates, as well as in-hospital complications, were comparable between sexes, reflecting the safety and feasibility of CTO-PCI in the female population; (3) unadjusted analysis showed higher rates of long-term MACE in women; and (4) after IPTW-adjustment, female sex was no longer associated with the primary endpoint, although it remained significantly associated with a nearly 3-fold increased risk of MI during long-term follow-up.

In accordance with contemporary literature, fewer women than men were referred for CTO-PCI in our cohort [15,16]. This lower referral rate may reflect both diagnostic and therapeutic biases [17,18], as women typically present at older ages, with atypical symptoms and a higher burden of hypertension and diabetes mellitus [19]. This clustering of factors likely contributes to referral bias, whereby less comorbid women are managed conservatively despite having less complex CTO morphology [5]. In this context, angiographic analysis demonstrated that women exhibited more favourable anatomical characteristics, including shorter lesions, fewer bifurcations, and less severe calcification, resulting in significantly lower J-CTO scores compared with men. Conversely, men more frequently required complex procedural strategies, such as femoral access, greater total stent length, and more frequent use of retrograde crossing techniques and IVUS, which likely contributed to longer procedures, higher contrast volumes, and greater radiation exposure. These findings are consistent with previous international registries [19,20]. Taken together, these data suggest that women undergoing CTO-PCI constitute a clinically more fragile yet anatomically more favourable subgroup, in which patient selection and operator strategy (rather than lesion complexity per se) may play a pivotal role in achieving comparable outcomes between sexes.

Regarding procedural success, contemporary literature exhibits significant sex disparities in outcomes. Large-scale registries, such as ERCTO and PROGRESS-CTO, have reported higher success rates among women, which are primarily attributed to their lower anatomical complexity [7,21]. Conversely, other studies have found no significant sex-based differences in procedural efficacy [17,19,20]. A similar inconsistency is observed in periprocedural complications; while female sex has been associated with higher rates of vascular access-site complications and bleeding in several registries (a finding that may appear counterintuitive given the lower anatomical complexity and shorter procedure times), it remains consistent with the higher burden of comorbidities and smaller vessel diameters typically found in this group [8,9,18,19,20,22]. Notably, in our cohort, both procedural success and complication rates were comparable between sexes, suggesting that in high-volume centres, the reported higher risk in female patients might be mitigated.

Regarding long-term outcomes, unadjusted analyses indicated higher MACE in women, largely driven by their adverse baseline profile. After IPTW adjustment, this association disappeared, except for MI, which remained significantly more frequent in women. This may be explained by their greater systemic atherosclerotic burden, older age, and higher prevalence of metabolic comorbidities. Beyond these factors, residual ischemia from microvascular dysfunction, progression of non-culprit disease, or differences in secondary prevention and medication adherence could also contribute. Notably, prior studies have highlighted less intensive lipid-lowering therapy among women despite similar cardiovascular risk [23,24]. Although specific data on this phenomenon in patients undergoing CTO-PCI are lacking, these findings highlight the need for future studies evaluating the impact of dyslipidaemia management and other secondary prevention strategies in both sexes in this context.

Current evidence on sex-specific outcomes after CTO-PCI is heterogeneous. Some cohorts have reported comparable MACE rates, but these studies often had short follow-up periods or limited sample sizes, potentially underpowering the detection of differences in ‘hard’ endpoints [25,26]. Our observation of increased MI in women aligns with prior registries suggesting higher vulnerability to ischemic recurrences despite similar procedural success rates [19]. Comparative analyses between PCI and optimal medical therapy (OMT) indicate sex-related interactions: Guo et al. reported that the reduction in MACE with CTO-PCI versus OMT was observed only in men, while Flores-Umanzor et al. found higher adverse event rates in women regardless of treatment strategy [5,27]. Taken together with the unfavourable risk profile of our female cohort, these data suggest that a conservative approach may be more frequently considered in selected female patients.

Evidence on very long-term outcomes remains limited. Only one previous prospective single-centre registry with an up to 8-year follow-up has addressed the sex-specific effects of procedural success in CTO-PCI, finding that successful recanalization reduced MACE irrespective of sex and sex did not independently predict procedural success [19,25]. Our findings are consistent, showing comparable technical success between men and women despite the more complex clinical profile in the female cohort. However, women still experienced a higher incidence of MI during follow-up, suggesting that while CTO-PCI feasibility is similar across sexes, the greater baseline comorbidity burden in women may drive residual ischemic risk over time. In contrast, men, even after complex procedures, did not exhibit excess ischemic events, supporting the long-term safety of CTO-PCI in experienced centres. Nevertheless, these findings should be interpreted with caution given the modest number of events, as reflected by the wide confidence intervals.

This study has several limitations related to its observational, retrospective, and single-centre design. Although the IPTW provides an adequate balance between measured confounders, the presence of unmeasured confounding cannot be entirely excluded. Furthermore, the single-centre nature of the registry may limit the generalizability of these findings to other populations or centres with different clinical practices or lower procedure volumes. A key limitation is that the relatively low proportion of women, although consistent with international CTO registries, might have limited the statistical power to detect differences in less frequent endpoints such as stroke. Detailed information on long-term medical therapy for both sexes was not available, which limits the interpretation of potential differences in long-term clinical outcomes.

## 5. Conclusions

In this contemporary, single-centre registry, women undergoing CTO-PCI tended to present with a more adverse clinical profile, despite exhibiting lower angiographic complexity compared with men. Technical and procedural success rates, as well as in-hospital complication rates, were comparable between sexes. Although long-term adjusted MACE rates appeared similar, a higher incidence of MI during follow-up was observed in women. Given the limited number of female patients, these findings should be interpreted within the context of the study design and warrant confirmation in larger, prospective multicentre cohorts.

## Figures and Tables

**Table 1:** Baseline characteristics.

	Overall (*n* = 928)	Female Patients (*n* = 140, 15%)	Male Patients (*n* = 788, 85%)	*p*
Age (years)	65.0 ± 10.2	69.7 ± 10.0	64.1 ± 10.0	<0.001
Smoking	295 (31.8%)	28 (20.3%)	266 (33.8%)	0.002
Hypertension	576 (62.1%)	104 (74.3%)	473 (60.0%)	0.002
Diabetes Mellitus	427 (46.0%)	83 (59.0%)	344 (43.7%)	<0.001
Dyslipidemia	575 (62.0%)	88 (62.9%)	487 (61.8%)	0.887
Atrial Fibrillation	97 (10.4%)	14 (9.9%)	83 (10.5%)	0.968
COPD	82 (8.8%)	6 (4.3%)	78 (9.9%)	0.048
Previous Stroke	43 (4.6%)	6 (4.3%)	36 (4.6%)	1.000
Peripheral Artery Disease	90 (9.7%)	9 (6.4%)	82 (10.4%)	0.192
Previous CAD	358 (38.6%)	41 (29.0%)	318 (40.4%)	0.017
Previous PCI	368 (39.7%)	43 (30.4%)	325 (41.2%)	0.024
Previous CABG	38 (4.1%)	1 (0.9%)	36 (4.6%)	0.056
LVEF (%) (mean, SD)	52.6 ± 12.2	55.9 ± 11.1	52.0 ± 12.3	0.001
eGFR (mL/min/1.73 m^2^) (mean, SD)	83.7 ± 32.7	79.7 ± 37.3	84.6 ± 31.5	0.108
NYHA III–IV	101 (10.9%)	20 (14.3%)	80 (10.1%)	0.192
CCS III–IV	252 (27.2%)	32 (23.2%)	221 (28.0%)	0.243
STEMI	129 (13.9%)	11 (7.6%)	118 (15.0%)	0.024
NSTEMI	380 (41.0%)	58 (41.7%)	322 (40.9%)	0.932
Chronic Coronary Syndrome	363 (39.0%)	58 (41.7%)	305 (38.5%)	0.547
Heart failure	56 (6.0%)	13 (9.1%)	43 (5.4%)	0.119

CABG: Coronary artery bypass grafting CAD: Coronary artery disease. COPD: Chronic obstructive pulmonary disease. CCS: Canadian Cardiovacular Society angina. eGFR: Estimated glomerutal filtration rate LVEF: Left ventricular ejection fraction NSTEMI: Non-ST-segment elevation myocardial infarction NYHA: New York Heart Association. PCI: Percutaneous coronary intervention. SD: Standard deviation. STEMI: ST-segment elevation myocardial infarction.

**Table 2:** Angiographic and procedural characteristics.

	Overall (*n* = 1003)	Female Patients (*n* = 146, 15%)	Male Patients (*n* = 857, 85%)	*p*
Multivessel Disease	96 (68%)	46 (68%)	50 (69%)	0.856
Syntax Score (median, IQR)	16.0 (12.0–21.0)	18.0 (14.0–21.5)	16.0 (11.3–20.8)	0.187
Target Vessel				
LAD	297 (29.6%)	50 (34.3%)	247 (28.8%)	0.219
LCx	214 (21.3%)	24 (16.4%)	190 (22.2%)	0.146
RCA	492 (49.0%)	72 (49.3%)	420 (49.0%)	1.000
Ostial Location	146 (14.6%)	26 (17.9%)	121 (14.1%)	0.299
Blunt	517 (51.5%)	72 (49.6%)	444 (51.8%)	0.640
Ambiguous Cap	398 (39.7%)	55 (37.5%)	342 (39.9%)	0.675
Bifurcation CTO	322 (32.1%)	34 (23.3%)	288 (33.6%)	0.018
Lesion Length (median, IQR)	33.1 (20.7–50)	25 (18–38)	35 (21–50)	<0.001
CTO Length (median, IQR)	21 (15–32)	18 (13–25)	22 (16–34)	<0.001
Lesion Length > 20 mm	585 (58.3%)	74 (50.8%)	511 (59.6%)	0.053
Bending > 45°	183 (18.2%)	21 (14.3%)	161 (18.8%)	0.246
Severe Tortuosity	148 (14.8%)	13 (8.7%)	135 (15.8%)	0.042
Severe Calcification	567 (56.5%)	69 (47.2%)	498 (58.1%)	0.019
Reattempt	179 (17.8%)	12 (8.5%)	166 (19.4%)	0.002
J-CTO Score (median, IQR)	2 (1–3)	2 (1–2)	2 (1–3)	<0.001
Intracoronary Imaging				
IVUS	242 (24.1%)	24 (16.4%)	218 (25.4%)	0.025
OCT	1 (0.1%)	0 (0.0%)	1 (0.1%)	1.00
Plaque Modification				
HPB	23 (2.3%)	5 (3.2%)	19 (2.2%)	0.233
ELCA	12 (1.2%)	2 (1.4%)	10 (1.2%)	0.377
Rotational Atherectomy	22 (2.2%)	1 (0.7%)	21 (2.5%)	0.690
Technical Approach				
AWE	698 (69.6%)	120 (82.5%)	578 (67.4%)	<0.001
ADR	80 (8.0%)	8 (5.8%)	72 (8.4%)	0.299
RWE	103 (10.3%)	7 (5.0%)	96 (11.2%)	0.027
RDR	121 (12.1%)	10 (6.7%)	111 (13.0%)	0.051
Stent Diameter (mm) (median, IQR)	3 (2.5–3)	3 (2.5–3)	3 (2.5–3.5)	0.048
Total Stent Length (mm) (median, IQR)	41 (27–60)	36 (24–48)	41.5 (28–61)	0.002
Main Femoral Access	694 (69.2%)	72(49.3%)	622 (72.6%)	<0.001
Procedural Time (min) (mean, SD)	129.1 ± 76.1	101.8 ± 48.3	134.7 ± 79.6	<0.001
Contrast Volume (mL) (mean, SD)	249.9 ± 118.8	234.7 ± 154.4	252.7 ± 111.1	0.014
Air Kerma (mGy) (median, IQR)	2189 (1301–3391)	1592 (1049–2810)	2297 (1395–3454)	0.001
Technical success	868 (86.5%)	125 (85.4%)	743 (86.7%)	0.695
Procedural success	866 (86.3%)	125 (85.4%)	741 (86.5%)	0.794

AWE: Antegrade wire escalation. ADR: Antegrade dissection and re-entry. CTO: Chronic total occlusion. ELCA: Excimer laser. IQR: Interquartile range. HPB: High-pressure balloon. IVUS: Intravascular ultrasound. OCT: optical coherence tomography RWE: Retrograde wire escalation. RDR: Retrograde dissection and re-entry. SD: Standard deviation.

**Table 3:** In-Hospital Complications.

	Overall (*n* = 928)	Female Patients (*n* = 140, 15%)	Male Patients (*n* = 788, 85%)	*p*
In-hospital death	2 (0.2%)	1 (0.7%)	1 (0.1%)	0.270
In-hospital stroke	1 (0.1%)	1 (0.7%)	0 (0.0%)	0.146
Periprocedural MI	1 (0.1%)	0 (0.0%)	1 (0.1%)	1.000
Major bleeding	4 (0.4%)	1 (0.7%)	3 (0.4%)	0.468
Major vascular complication	4 (0.4%)	0 (0.0%)	4 (0.5%)	1.000
Minor vascular complication	2 (0.2%)	1 (0.7%)	1 (0.1%)	0.270
Coronary perforation	10 (1.0%)	2 (1.4%)	9 (1.0%)	0.667
Collateral complication	6 (0.6%)	1 (0.7%)	5 (0.6%)	1.000
Cardiac tamponade	6 (0.6%)	2 (1.4%)	4 (0.5%)	0.213
AKI	20 (2.0%)	3 (2.1%)	17 (2.0%)	1.000

AKI: Acute kidney injury. MI: Myocardial infarction.

**Table 4:** Baseline characteristics. Clinical outcomes.

Endpoint	No. Events (%)		Estimate (95% CI)	*p*
	Male Patients	Female Patients		
**Primary endpoint**				
All-cause death, MI, or stroke	174 (22.1%)	41 (29.3%)	HR: 1.51 (1.08–2.13)	0.017
			aHR: 1.15 (0.76–1.74)	0.517
**Secondary endpoints**				
All-cause death, MI, stroke, or clinically driven revascularization	260 (33.0%)	53 (37.9%)	HR: 1.29 (0.96–1.73)	0.091
			aHR: 1.18 (0.82–1.70)	0.369
All-cause death	154 (19.5%)	31 (22.1%)	HR: 1.24 (0.84–1.82)	0.275
			aHR: 0.90 (0.57–1.41)	0.646
MI	23 (2.9%)	11 (7.9%)	HR: 2.90 (1.41–5.96)	0.004
			aHR: 2.85 (1.23–6.63)	0.015
Stroke	16 (2.0%)	7 (5.0%)	HR: 2.74 (1.13–6.66)	0.026
			aHR: 1.95 (0.71–5.33)	0.193
Clinically driven revascularization	108 (13.7%)	23 (16.4%)	HR: 1.28 (0.82–2.01)	0.281
			aHR: 1.50 (0.88–2.55)	0.140

aHR: adjusted HR. CI: confidence interval. HR: hazard ratio. MI: myocardial infarction. HRs of non–fatal endpoints correspond to cause specific HR.

## Data Availability

The de-identified data supporting the findings of this study, as well as the analysis code used to generate the results, are available from the corresponding author upon reasonable request.

## Supplementary Materials

The following supporting information can be downloaded at https://www.mdpi.com/article/10.3390/jcm15041449/s1: Figure S1: Propensity Score distribution before and after weighting; Figure S2. Covariate balance across the 20 imputed datasets; Table S1: Sensitivity Analysis for Non-Fatal Endpoints Using Fine-Gray Competing Risk Models; Table S2: Clinical endpoints IPW Cox models using multiple imputation.

## Abbreviations

The following abbreviations are used in this manuscript:

ACS	Acute coronary syndrome
ADR	Antegrade dissection and re-entry
aHR	Adjusted hazard ratio
AKI	Acute kidney injury
AWE	Antegrade wire escalation
BARC	Bleeding Academic Research Consortium
CABG	Coronary artery bypass grafting
CAD	Coronary artery disease
CCS	Canadian Cardiovascular Society
CI	Confidence interval
COPD	Chronic obstructive pulmonary disease
CTO	Chronic total occlusion
CTO-ARC	Chronic total occlusion Academic Research Consortium
eGFR	Estimated glomerular filtration rate
ELCA	Excimer laser
HPB	High-pressure balloon
HR	Hazard ratio
IPTW	Inverse Probability of Treatment Weighting
IQR	Interquartile range
IVUS	Intravascular ultrasound
J-CTO	Multicentre CTO Registry in Japan
LAD	Left anterior descending artery
LCx	Left circumflex artery
LVEF	Left ventricular ejection fraction
MACE	Major adverse cardiovascular events
MI	Myocardial infarction
NSTEMI	Non-ST-segment elevation myocardial infarction
NYHA	New York Heart Association
OCT	Optical coherence tomography
OMT	Optimal medical therapy
PCI	Percutaneous coronary intervention
RCA	Right coronary artery
RDR	Retrograde dissection and re-entry
RWE	Retrograde wire escalation
SD	Standard deviation
SMD	Standardized Mean Difference
STEMI	ST-segment elevation myocardial infarction
TIMI	Thrombolysis In Myocardial Infarction
