# Supplementary material for: Sex-Based Differences in Long-Term Outcomes Following Percutaneous Coronary Intervention for Chronic Total Occlusions
Source: J Clin Med. 2026 Feb 12;15(4):1449. doi: 10.3390/jcm15041449 (PMC12941348; doi:10.3390/jcm15041449)
Supplement: Supplementary file 1 [file jcm-15-01449-s001.zip › jcm-4113955-supplementary.pdf]

SUPPLEMENTARY MATERIALS

Supplementary Figures

1. Figure S1:

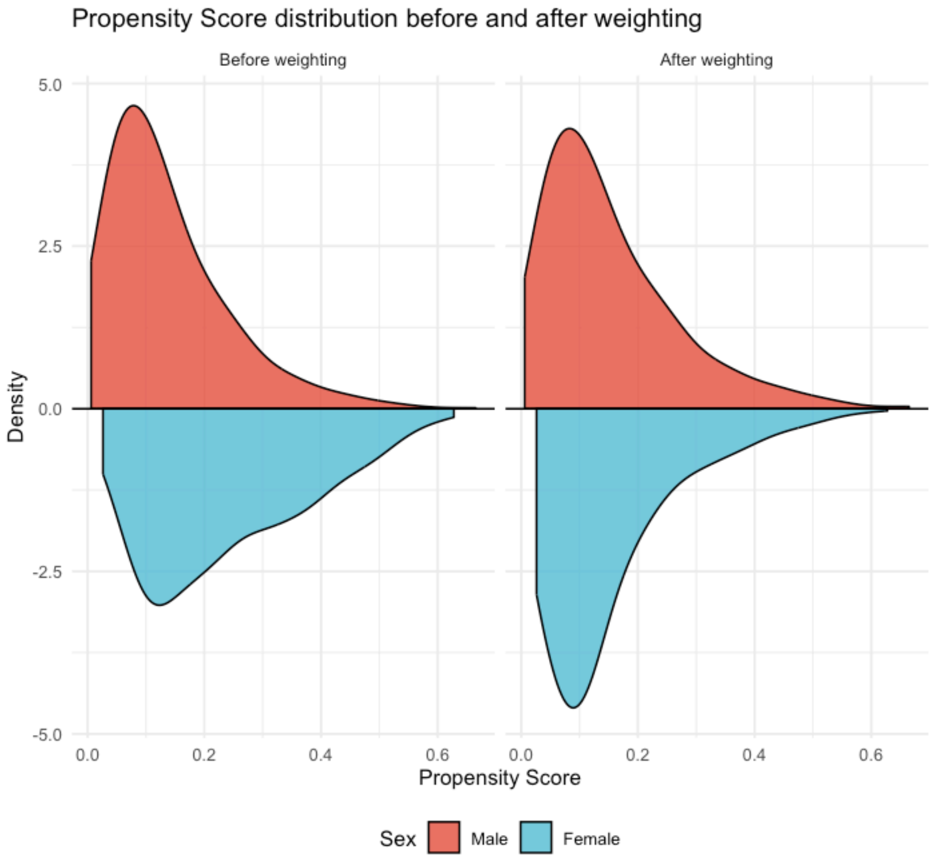

Figure S1. Propensity Score distribution before and after weighting.

2. Figure S2:

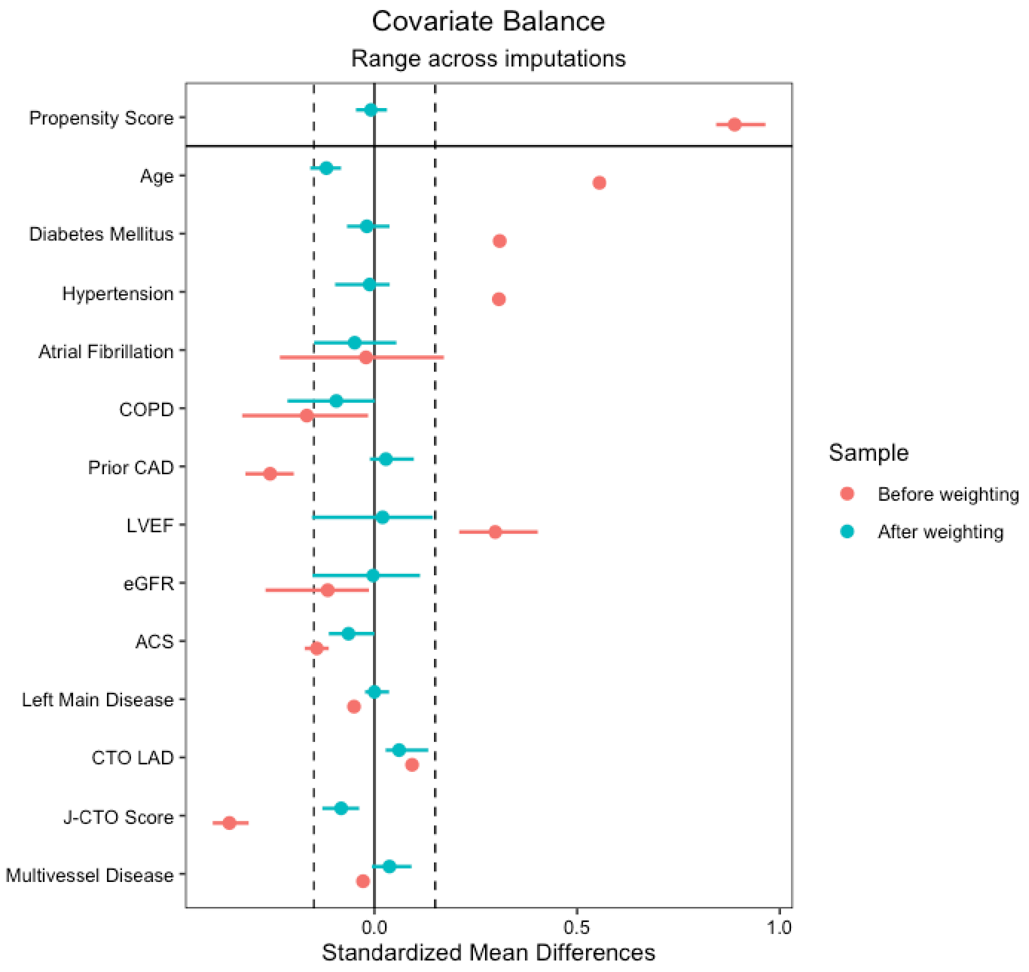

Figure S2. Covariate balance across the 20 imputed datasets. ACS: acute coronary syndrome; CAD: coronary artery disease; COPD: chronic obstructive pulmonary disease; CTO: chronic total occlusion, eGFR: estimated glomerular filtration rate; LAD: left anterior descending artery; LVEF: left ventricular ejection fraction.

Suplementary Tables

1. Table S1:

Table S1 Sensitivity Analysis for Non-Fatal Endpoints Using Fine-Gray Competing Risk Models.

Endpoint	Model	sHR (95% CI)	*P*
Myocardial Infarction	Unadjusted	2.86 (1.39–5.86)	0.004
	IPW Adjusted	2.88 (1.24–6.68)	0.014
Stroke	Unadjusted	2.66 (1.09–6.47)	0.031
	IPW Adjusted	1.96 (0.72–5.36)	0.189
Revascularization	Unadjusted	1.26 (0.80–1.98)	0.310
	IPW Adjusted	1.51 (0.89–2.57)	0.127

CI: confidence interval. sHR: subdistribution hazard ratio. All-cause death was considered a competing risk for all non-fatal endpoints. sHRs were calculated using Fine-Gray competing risk models. Adjusted models were

performed using Inverse Probability of Treatment Weighting (IPTW) based on the propensity score

1. Table S2:

Table S2. Clinical endpoints IPW Cox models using multiple imputation

Endpoint	aHR (95% CI)	*P*
All-cause death, MI or stroke	1.21 (0.77-1.91)	0.412
All-cause death, MI, stroke or clinically driven revascularization	1.85 (0.85-1.85)	0.243
All–cause death	0.93 (0.56-1.54)	0.777
Myocardial infarction	3.07 (1.20-7.86)	0.021
Stroke	1.97 (0.62-6.26)	0.232
Clinically driven revascularization	1.59 (0.91-2.79	0.101

MI: Myocardial infarction. aHR: ajusted (IPW) Hazard ratio. CI: Confidence Interval
